# Immediate and short-term effects of the combination of dry needling and percutaneous TENS on post-needling soreness in patients with chronic myofascial neck pain

**DOI:** 10.1590/bjpt-rbf.2014.0176

**Published:** 2016-07-11

**Authors:** Jose V. León-Hernández, Aitor Martín-Pintado-Zugasti, Laura G. Frutos, Isabel M. Alguacil-Diego, Ana I. de la Llave-Rincón, Josue Fernandez-Carnero

**Affiliations:** 1 Department of Physical Therapy, Faculty of Health Science, The Center for Advanced Studies University La Salle, Universidad Autónoma de Madrid, Aravaca, Madrid, Spain; 2 Research Group on Movement and Behavioural Science and Study of Pain, The Center for Advanced Studies University La Salle, Universidad Autónoma de Madrid, Aravaca, Madrid, Spain; 3 Department of Physical Therapy, Faculty of Medicine, Universidad CEU San Pablo, Madrid, Spain; 4 Department of Physical Therapy, Occupational Therapy, Rehabilitation and Physical Medicine, Universidad Rey Juan Carlos, Madrid, Spain; 5 Hospital La Paz Institute for Health Research, IdiPAZ, Madrid, Spain

**Keywords:** neck pain, disability, trigger points, TENS, physical therapy

## Abstract

**Background:**

Dry needling (DN) and percutaneous electrical nerve stimulation (PENS) are widely used techniques in the treatment of myofascial pain.

**Objective:**

To investigate the immediate and short-term effects of the combination of DN and PENS compared to DN alone on the upper trapezius muscle.

**Method:**

This is a 72-hour follow-up single-blinded randomized controlled trial. Sixty-two volunteer patients with chronic myofascial neck pain with active Myofascial Trigger Points (MTrPs) in the upper trapezius muscle were recruited. Randomization was performed, and 31 patients received DN treatment (DN group) and 31 received DN and PENS (DN+PENS group). The primary outcomes were neck disability index (NDI) and visual analog scale for pain for both post-needling soreness (PNS) and neck pain intensity (NPI). Pressure pain threshold (PPT) and cervical range of motion (CROM) were the secondary outcomes.

**Results:**

We detected between-group differences in NPI and PNS in favor of the DN+PENS group immediately after treatment. No between-group differences in NDI were observed.

**Conclusion:**

PENS application after dry needling treatment is more effective than dry needling alone for decreasing soreness in the short term and improving neck pain intensity immediately in patients with myofascial chronic neck pain.

## BULLET POINTS

- The application of PENS after DN to active myofascial trigger points shows higher hypoalgesic effects than DN alone, reducing post-needling soreness immediately and in a short-term follow-up. There was no difference in terms of disability.
- The combination of PENS and DN has an immediate hypoalgesic effect, reducing neck pain intensity in myofascial chronic neck pain patients. However, both DN and the combination of DN and PENS reduce neck pain in a short-term follow-up.
- Given that both DN and the combination of DN and PENS decrease neck pain, it is recommended that the combination (DN+PENS) be applied in order to avoid post-needling soreness, which is the most common secondary side effect.

## Introduction

Myofascial pain is commonly defined as a frequent source of pain in clinical practice. Myofascial pain is a prevalent pathology in developed countries, with epidemiological studies showing that up to 85% of the general population will experience at least one episode of myofascial pain during their lifetime^1^.

Myofascial trigger points (MTrPs) are often present in this pain-related pathology and are defined as hypersensitive spots located in the palpable taut bands of skeletal muscle^2^. MTrPs can be painful on compression and can produce characteristic effects, such as alteration of muscle activation^3^, increased muscle tension, restricted range of motion, muscle weakness^4^, fatigability^5^, and autonomic phenomena^6^. MTrPs can be classified as active or latent. Active MTrPs produce local or referred spontaneous pain that can be elicited by stimulation. Latent MTrPs produce local or referred pain only when stimulated, but not spontaneously^2^ ^,^ ^6^.

Many non-pharmacological techniques are applied for the treatment of MTrP worldwide^7^. Among them, electrotherapy has been widely used as a hypoalgesic agent, which typically involves transcutaneous nerve stimulation (TENS)^8^ ^,^ ^9^. Another well-known hypoalgesic technique is dry needling (DN)^10^ . This kind of puncture has been shown to have a similar efficacy at alleviating musculoskeletal pain as lidocaine injection^11^. DN is an invasive technique that involves the introduction of a needle into the muscle, directed at the MTrP. DN has been associated with secondary side effects, which include the appearance of post-needling soreness^12^. The duration of this soreness is thought to vary from a few hours to 2-3 days when solid filament needles are used^13^ ^,^ ^14^. This side effect can lead to patient dissatisfaction, patients developing an aversion to the technique, and subsequently a loss of treatment adherence^12^. Therefore, minimizing potential soreness resulting from DN could be a goal of therapists who use dry needling.

Additional techniques have been combined with DN to inhibit MTrPs and reduce post-needling soreness. For example, ischemic compression^15^, ‘spray and stretch’^14^, and ultrasound^12^ have been applied with positive effects, reducing both myofascial pain^16^ and post-needling soreness^12^ ^,^ ^14^ ^,^ ^15^. According to recent studies, TENS operates via multiple pathways to reduce pain through various physiological mechanisms^17^ ^,^ ^18^. Furthermore, many studies have shown large hypoalgesic effects when TENS is applied using an electroacupuncture approach, in which needles are used as electrodes to avoid skin impedance^19^ ^-^ ^21^. This modality is known as percutaneous electrical nerve stimulation (PENS). Using PENS, large hypoalgesic effects have been reported in animal pain models^22^ ^,^ ^23^. To our knowledge, however, the effectiveness of PENS applied immediately after dry needling for the treatment of post-needling soreness has not been previously investigated.

The objective of this study was to assess the effectiveness of a combined treatment using PENS with DN approach, versus DN alone, on improving post-needling soreness, neck pain, neck disability, pressure pain threshold (PPT), and cervical range of motion (CROM) in chronic neck pain patients with active upper trapezius MTrPs.

## Method

This is a single-blinded randomized controlled trial. The outcome assessor did not know the group to which the subject was allocated. Sample size calculation were performed using G*Power software version 3.1.7 (Heinrich-Heine-Universität, Düsseldorf, Germany)^24^. Considering an effect size of 0.25, a minimum power of 0.95, and α value of 0.05, the estimated sample size was 44 subjects. Allowing for a conservative dropout of 20%, we planned to recruit 54 subjects, 27 to for each group. Sixty-five subjects with chronic nonspecific neck pain were screened for possible eligibility criteria. Finally, sixty-two patients with chronic neck pain (16 men, 46 women) aged 18 to 48 years (mean [SD], 25 [8] years old) met the inclusion criteria for this study. Inclusion criteria were: (1) neck pain for more than 6 months; (2) neck pain of at least 3 cm on a 0-10 cm visual analog scale (VAS); (3) presence of a palpable taut band in the muscle; (4) presence of a hypersensitive tender spot in the taut band; (5) palpable or visible local twitch response (LTR) with snapping palpation of the taut band; and (6) local or referred neck pain elicitation in response to compression. These criteria had good interexaminer reliability (κ), ranging from 0.84 to 0.88^25^.

Participants were excluded if they presented any of the following criteria: (1) previous treatment of DN; (2) radiculopathies and/or radicular pain; (3) whiplash-related neck pain; (4) dizziness; (5) migraines; (6) previous cervical surgical intervention; or (7) diagnosis of fibromyalgia.

A blinded researcher performed the randomization of subjects, using the statistical program GraphPad (GraphPad Software, Inc. La Jolla, CA, USA), obtaining two blocks of 31 subjects, corresponding to each group of treatment. Only the therapist had access to the allocation schedule. Due to the nature of the interventions, both patients and treatment provider were not blinded to the treatment allocation.

This trial was approved by the Ethical Committee of Rey Juan Carlos University, Madrid, Spain, with identification number 50/2012. This study was also registered under number NCT02230709. All subjects signed an informed consent form before their inclusion.

### Outcome measures

The primary outcomes were pain intensity for post-needling soreness and neck pain intensity as well as disability. All other outcomes were considered secondary.

Using a pain diary, post-needling soreness was measured at 24, 48, and 72 h post-treatment. Neck pain intensity was measured before DN, immediately after DN and at 72 h post-treatment. The degree of disability was registered before DN and at 72 h post-treatment. PPT and CROM were assessed before DN, immediately after DN, and at 72 h post-treatment.

A VAS scale was used for measuring pain intensity. This scale consisted of a 10 cm long line, where the 0 cm point corresponds to “no pain” and the 10 cm point corresponds to the “worst imaginable pain”. The patients placed a vertical mark corresponding to their level of pain. The Numerical Rating Scale (NRS) may also be used to measure pain score. Both methods have been demonstrated to be reliable and valid instruments for the measurement of neck pain^26^, with a change of 2 cm (or 2 points) being described as “much improved” in chronic pain patients^27^.

Post-needling soreness was quantified using a pain diary with a visual analog scale. The patient completed the diary by registering in the VAS the pain intensity in the needled area four times per day (in the morning, before lunch, in the afternoon, and in the evening) during the three days following treatment. Subjects were asked to specifically rate the post-needling soreness separately from the original myofascial neck pain.

The validated Spanish version of the Neck Disability Index (NDI) was used to assess the degree of disability. The NDI is a valid tool for the measurement of pain and self-assessment of cervical disability. The NDI is composed of 10 questions related to daily functional activities. NDI presents an acceptable reliability with an intraclass correlation coefficient (ICC) ranging from 0.50 to 0.98^28^.

Pressure Pain Threshold (PPT) was assessed using a digital algometer (Wagner Instruments, Greenwich, CT, USA) that reported measurements in kg/cm^2^. PPT is defined as the minimal amount of pressure that induces pain. A physical therapist with 3 years of experience in algometry took three measurements at 30-sec intervals; the mean of the three measurements was used in subsequent analyses. PPT presents high interexaminer reliability with an intraclass correlation coefficient (ICC) of 0.91 and high intraexaminer reliability (ICC=0.94-0.97) in the upper trapezius muscle^29^.

Cervical Range of Motion (CROM) was measured using a cervical goniometer (Performance Attainment Associates, St. Paul, MN, USA). Subjects performed neck movements to the fullest extent of their mobility of flexion, extension, right and left lateroflexion, and right and left rotation. The mean of three measurements was calculated for each movement. CROM presented a good intraexaminer and interexaminer reliability with a ICC>0.80^30^. Furthermore, it has proven to be a validated method for measuring cervical range of motion^31^ ^,^ ^32^.

### Procedure

Subjects in the first group (DN group) received deep DN to the upper trapezius active MTrP until two local twitch responses were elicited. The physical therapist previously disinfected the area and performed deep DN based on the method described by Hong^11^, in which several manipulations of an acupuncture needle (0.32 × 40 mm, Suzhou Huanqiu Acupuncture Medical Appliance Co. Ltd., Suzhou, Jiangsu, China) are performed by quickly inserting and partially withdrawing the needle. The eliciting of local twitch responses is related to a greater effectiveness of the technique^11^. Finally, the therapist removed the needle and compressed the area for 90 sec.

Subjects in the second group (DN+PENS group) received deep DN to the upper trapezius active MTrP until two local twitch responses were elicited and PENS was applied immediately after, using a portable TENS device (model TN-20, EasyMed Instrument Co Ltd., Foshan, China) with the needle as the negative electrode. A second electrode was an adhesive one, and it was placed 1 cm lateral to the positive electrode. The parameters used were a compensated symmetrical pulsed biphasic current of low frequency (2 Hz) and a pulse width of 120 μs applied over a 15 min period. The patient was asked to indicate when the current intensity was well tolerated and not painful.

### Data analysis

The statistical analyses were carried out using the SPSS statistical software system version 20.0 (SPSS Inc., Chicago, IL, USA). Using the Kolmogorov-Smirnov test, CROM was found to be normally distributed, whereas VAS, PPT, post-needling soreness, and NDI variables were not normally distributed. Descriptive statistics were used to summarize data, including means and SDs, medians, and interquartile ranges for continuous data. A 2×3 repeated measures ANOVA was performed to evaluate the effect of the CROM variable with intervention (i.e., the DN or DN+PENS Group) and time (pretreatment, immediately post-treatment, and 72 h post-treatment) as factors. Tests of within-patients post hoc sample effects (i.e., changes in time for all variables for each group separately) were performed with Bonferroni corrections. The Mann-Whitney U test was used for the analysis of the NDI variable. For the remaining variables, between-group differences were assessed by the Kruskal-Wallis test. The Wilcoxon signed rank test was used for post hoc within-group comparisons. Statistical tests were interpreted at the 5% significance level. The analyses followed the intention-to-treat principles.

### Results

**Figure 1:**
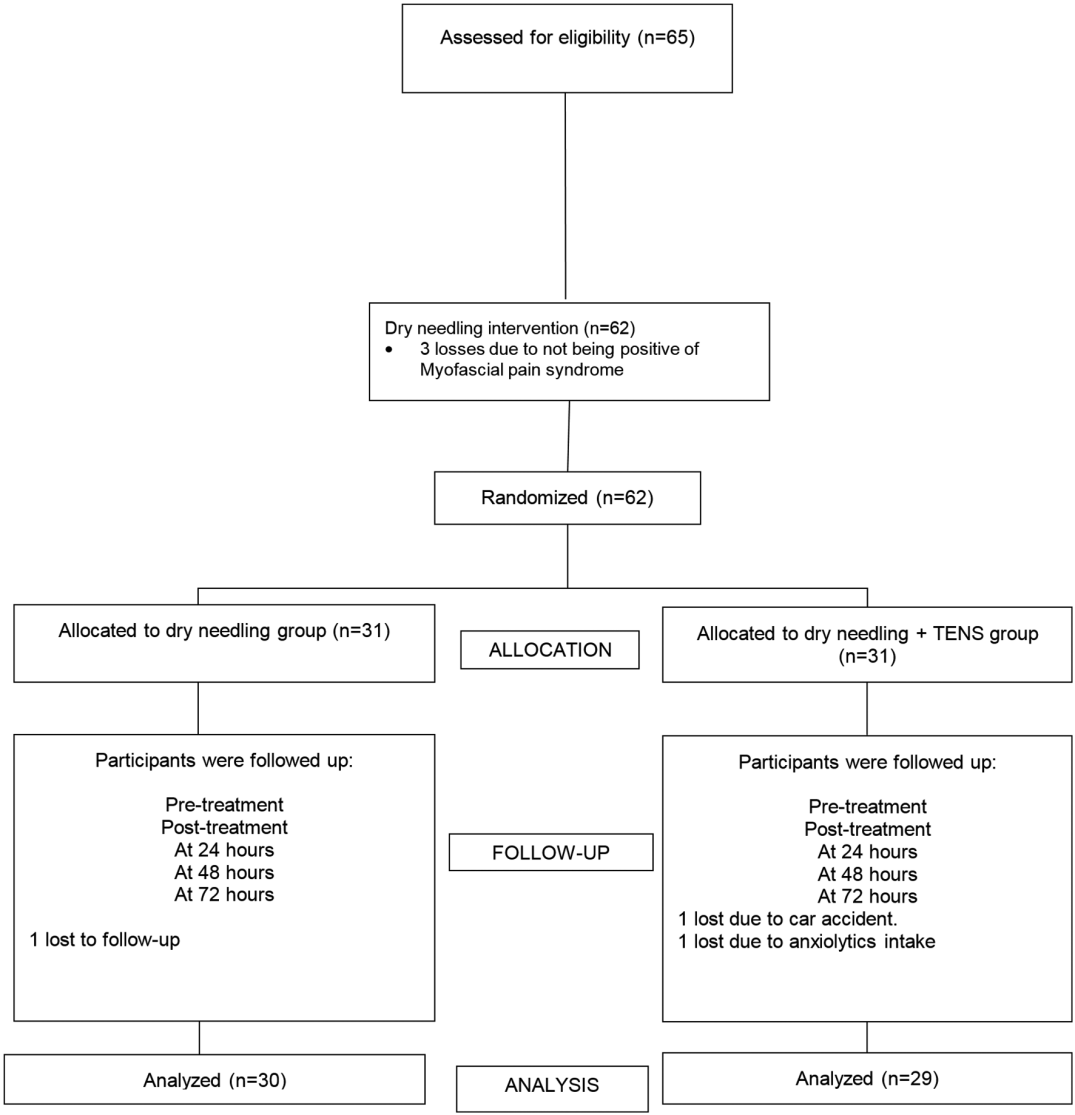
Flow diagram.

Sixty-two patients successfully completed the study protocol, of which 31 were randomly assigned to the DN Group (7 men, 24 women; mean age [SD], 23 [5] y) and 31 were assigned to the DN+PENS Group (9 men, 22 women; mean age [SD], 27 [10] y). A total of 30 subjects were analyzed in the DN Group and 29 subjects were analyzed in the DN+PENS Group (Figure 1).

The patients’ demographic and clinical characteristics at the beginning of the study are summarized in Table 1.

**Table 1:** Sample’s baseline characteristics.

**Outcomes Measures**	**DN (n=31)**	**DN+PENS (n=31)**
**Age years**	23.32±4.77	26.81±9.63
**Gender M/F (female%)**	7/24 (77.4%)	9/22 (71.0%)
**Neck pain duration (months)**	16.03±17.23	19.36±19.23
**VAS (0-100mm)**	4.82±1.91	4.82±1.83
**PPT (Kg/cm^2^)**	1.72±0.81	1.80±0.90
**Flexion (degrees)**	52.16±11.67	49.03±13.09
**Extension (degrees)**	58.41±12.75	56.41±12.96
**Right Lateral Flexion (degrees)**	38.71±10.08	37.34±8.96
**Left Lateral Flexion (degrees)**	40.63±9.23	37.44±9.57
**Right Rotation (degrees)**	60.18±9.70	59.56±10.80
**Left Rotation (degrees)**	61.03±12.10	59.94±13.00
**NDI**	10.09±4.07	11.04±4.70

Values are mean±SD. VAS: Visual Analog Scale - Neck pain intensity; PPT: Pressure pain threshold; NDI: Neck Disability Index; DN: Dry Needling.

### Primary outcomes

#### VAS score for post-needling soreness

For the between-group comparisons using the Kruskal-Wallis test, differences were found at all follow-up periods (P<.05), showing greater improvements in the DN+PENS group. The post-needling soreness findings are presented in Table 2.

**Table 2:** Non-parametric tests of outcome data. Post-needling soreness and Neck Pain Intensity.

			**Median (first and third quartiles)**				**Wilcoxon** **a) 24h vs. 48h** **b) 24h vs. 72h** **c) 48h vs. 72h**
		**Group**	**24h Post-DN**	**48h Post-DN**	**72h Post-DN**		
**PNS**		**DN**	5.00 (3.25 and 6.12)	3.50 (2.12 and 5.00)	3.00 (1.00 and 4.75)		**a) .002** **b) <.001** **c) .110**
		**DN + PENS**	2.75 (0.50 and 5.00)	1.00 (0.00 and 3.75)	1.00 (0.00 and 2.75)		**a) .038** **b) .002** **c) .005**
	**Kruskal-Wallis**		**.002**	**<.001**	**<.001**		
		**Group**	**Baseline**	**Immediately Post-DN**	**72h Post-DN**		**Wilcoxon** **a)Baseline vs. Post** **b)Baseline vs. 72 h** **c)Post vs. 72 h**
**VAS** **NPI**		**DN**	5.00 (4.00 and 6.00)	5.00 (3.00 and 6.25)	2.50 (1.00 and 4.00)		**a) .504** **b) <.001** **c) .001**
		**DN + PENS**	5.00 (3.50 and 6.00)	3.00 (1.25 and 4.50)	2.00 (1.00 and 5.00)		**a) .001** **b) .001** **c) .014**
	**Kruskal-Wallis**		.742	**.016**	.669		

PNS: Post-needling soreness; DN: Dry Needling; VAS-NPI: Visual Analog Scale - Neck Pain Intensity.

#### VAS score for neck pain intensity

In the between-group comparison using the Kruskal-Wallis test, differences were found in the immediately post-treatment measures (P<.05), showing a decrease in VAS scores for DN+PENS group (Table 2).

#### Neck disability

In the between-group comparisons (Mann-Whitney U test), no significant differences in NDI were found at 72 h post-treatment (P>.05) (Table 3).

**Table 3:** Non-parametric tests of outcome data for NDI and PPT.

			**Median (first and third quartiles)**				**Wilcoxon** **Baseline vs 72h post-DN**
		**Group**	**Baseline**		**72h Post-DN**		
**NDI**		**DN**	9.50 (8.00 and 13.00)		6.50 (3.25 and 10.00)		**.005**
		**DN + PENS**	11.00 (7.00 and 14.50)		6.00 (4.00 and 14.00)		**.005**
	**Kruskal-Wallis**		.588		.700		
		**Group**	**Baseline**	**Immediately Post-DN**	**72h Post-DN**		**Wilcoxon** **a)Baseline vs. Post** **b)Baseline vs. 72 h** **c)Post vs. 72 h**
**PPT**		**DN**	1.67 (1.00 and 2.00)	1.58 (1.03 and 1.99)	1.50 (1.15 and 1.87)		**a) .112** **b) .180** **c) .469**
		**DN + PENS**	1.66 (1.27 and 3.03)	2.43 (1.25 and 4.50)	1.70 (1.18 and 6.75)		**a) .016** **b) .927** **c) .280**
	**Kruskal-Wallis**		.577	.050	.371		

NDI: Neck Disability Index; DN: Dry Needling; PENS: Percutaneous electrical nerve stimulation; PPT: Pressure pain threshold.

### Secondary outcomes

#### Pressure pain threshold

Both groups presented no statistically significant differences in PPT between baseline and all follow-up periods (P>.05.). In the between-group comparisons (Kruskal-Wallis test), differences were found at the immediate post DN (P<.05), showing a higher improvement in the DN+PENS group (Table 3).

#### Cervical range of motion

Repeated measures ANOVA findings for CROM outcomes were as follows: The time × group interaction did not show statistical significant changes in any of the movements (P>.05 in all of them), so no differences between group were found. (Table 4).

**Table 4:** Cervical range of movement, expressed in degrees, over time.

**Mean ± SD (95%CI)**					
**Outcomes Measures**	**Group**	**Baseline**	**Post DN**	**72 h Post DN**	**Cohen’s d**
**FLEXION**	**DN** **DN + PENS**	52.42±11.72 52.05±12.24	49.96±13.71 53.24±12.53	51.07±12.21	.112
				53.76±12.07	.140
**EXTENSION**	**DN** **DN + PENS**	58.11±12.44 58.62±11.80	57.27±13.91 61.13±12.10	**60.26±13.73** *	.164
				**62.98±10.90** *	.383
**LEFT LATERO FLEXION**	**DN** **DN + PENS**	39.05±8.01 38.16±9.36	39.79±9.21 41.78±9.42	**39.77±9.18** *	.083
				**41.80±9.63** *	.383
**RIGHT LATEROFLEXION**	**DN** **DN + PENS**	38.77±10.46 37.55±9.11	36.94±8.93 40.00±11.23	**40.59±9.67** *	.180
				**40.75±9.91** *	.336
**LEFT ROTATION**	**DN** **DN + PENS**	61.40±12.82 59.31±13.72	62.48±9.98 63.69±9.30	64.33±8.58	.268
				59.59±11.98	.021
**RIGHT ROTATION**	**DN** **DN + PENS**	60.36±10.24 60.50±10.73	59.95±8.86 60.04±11.37	62.22±7.42	.208
				61.18±10.92	.062

DN: Dry Needling.

* P<.05 (within-group comparison, ANOVA).

## Discussion

This study has found that combined DN plus PENS treatment significantly outperformed DN alone at reducing post-needling soreness in the first 72 h after treatment. Moreover, after a single session of DN on the trapezius muscle, neck pain intensity was decreased at 72 h post-treatment, but not immediately after treatment. However, when PENS was included, neck pain intensity was decreased immediately post-treatment and at 72 h post-treatment. This reduction was significantly greater than the improvement produced by DN alone. Both groups improved in disability, regardless of whether PENS was applied. Only within-group differences were found in extension and lateroflexion in both groups at the 72 h follow-up.

### VAS score for post-needling soreness

The additional use of PENS immediately following DN resulted in a significantly greater reduction in post-needling soreness compared to DN alone^27^. To our knowledge, this is the first study assessing the effectiveness of additional therapy after DN for reducing post-needling soreness in active MTrPs. In the study of Lai^12^, the application of ultrasound after active trigger point injection has been shown to increase PPT and range of motion compared to patients who received trigger point injection without ultrasound.

Regarding latent MTrPs, recent studies have shown that the application of ‘spray and stretch’^14^ or ischemic compression^15^ after DN immediately decreased post-needling soreness compared to subjects who were only treated by DN. The use of DN plus PENS may have clinical relevance for professionals using DN in the treatment of MTrPs, since high post-needling soreness values are associated with patient dissatisfaction and reduced treatment adherence^12^.

### VAS score for neck pain intensity

In our study, patients in the DN+PENS group obtained greater improvements in neck pain than those who received DN alone. The immediate neck pain decrease in the DN+PENS group was 2.5 cm, which is greater than the minimum change accepted as likely to be clinically relevant (2 points or 2 cm)^27^. No immediate decrease was found in the DN group, and an improvement was found only at 72 h post-treatment, with a pain intensity decrease of 2.5 cm. Various studies have reported similar results in VAS scores with longer follow up. When a 1 week follow-up was performed, Mejuto-Vázquez et al.^33^ obtained similar changes, with scores between 1.9 cm (immediately) and 3.7 cm (1-week follow-up). Moreover, in the study of Llamas-Ramos et al.^34^, the improvement was higher at 2 weeks post-treatment, reaching VAS scores of 5.3 cm^34^. Other studies comparing various types^35^ and parameters^36^ of TENS have been carried out on chronic pain patients. These studies have found a pain decrease of between 0.93 and 1.4 cm. In these studies, the application of TENS was performed more intensely than in our study; however, our decrease in pain scores was greater (2.0 to 3.0 cm). Therefore, we can conclude that the application of DN plus PENS produces a higher magnitude of change than conventional TENS and that the hypoalgesic effect is due not only to the opioid mechanism triggered by PENS application^37^, but to serotonergic^38^ and adrenergic systems^18^. When only DN is performed, the main hypoalgesic effect is mediated by stimulation of the spinal dorsal horn, which blocks the nociceptive input. DN also stimulates opioid release, but this effect is not immediate^39^, so the pain-reducing effect is not as quick as that obtained when TENS is applied. When TENS is performed, spinal inhibition takes place following the “gate control” principle, leading to immediate hypoalgesia in addition to the opioid release triggered by the low frequency selected.

### Neck disability

Neck disability decreased significantly from baseline after the application of both DN and the combination of DN and PENS. No significant differences were found between-groups. These results are in line with previous DN studies that have reported functional improvements in myofascial pain patients^40^ ^-^ ^42^. A recent study found an improvement in disability assessed using the Disability of Arm, Hand, and Shoulder questionnaire after the application of DN to active MTrPs of the UT muscle^40^. Nevertheless, the NDI improvements shown in the present study are limited in relevance since they did not reach the minimum clinically important difference of 10 points reported by Young et al.^43^.

### Pressure pain threshold

Mechanical hypoalgesia was found immediately after treatment in the DN+PENS group, but not in the DN group, with the between-group differences being statistically significant in favor of the DN+PENS group.

The improvement in PPT scores after treatment in the DN+PENS group was of 0.77 Kg/cm^2^, which reached the minimal detectable change (defined as between 0.47 kg/cm^2^ and 1.2 kg/cm^2^)^44^.

These findings demonstrate that the application of PENS combined with the DN technique decreases the mechanical pain in the needled area. When only DN is performed, hypersensitivity is developed, so the PPT decreased among the DN group subjects. Recent studies using various frequencies of TENS^35^ ^,^ ^45^ and its combination with another physical agent (e.g., stretching, hot packs, ischemic compression, or ‘spray and strech’^46^) have shown significant improvements in PPT scores. Although these studies involved patients with latent trigger points and our subjects had active trigger points, the same results were obtained, suggesting that the application of PENS with DN is more effective than DN alone. This effect may be explained by the stimulation of opioid release triggered by PENS current^47^, which occurs in the treatment area. This is in contrast with the analgesic mechanism induced by the DN technique, which occurs mainly in the dorsal horn^48^. This particular type of analgesia can take several minutes to develop, so that no immediate effects were found when DN was performed, whereas immediate effects were seen when TENS was added to the treatment regime.

### Cervical range of motion

The application of PENS after DN did not significantly increase CROM, when compared to the DN group. A recent study assessed the effectiveness of MTrP lidocaine injection combined with ischemic compression (versus injection alone) in the treatment of active MTrPs in the trapezius muscle^16^. The authors reported an improvement in lateroflexion only when ischemic compressions were applied. The follow-up was 1 week after treatment. Although no statistically significant differences were found in our study, the results at 72 h were higher (3.2° versus 1.82°) when PENS was applied. According to our results, the addition of a complementary therapy to a needling technique did not significantly improve CROM compared to the needling group. In contrast, another study showed that the use of ultrasound after MTrP lidocaine injection in the trapezius muscle significantly improved CROM, when compared to injection alone^12^.

Regarding within-group analysis, only the DN+PENS group showed significant improvements in CROM from baseline in the extension and lateroflexion movements. However, these results are limited in relevance because the CROM improvements did not reach the minimum detectable change reported by Audette et al. for subjects with neck pain (5.1º for extension and 6.5º for flexion)^49^. These results are in contrast with previous research in which DN applied to an active UT muscle MTrP significantly improved CROM^11^ ^,^ ^33^. In our study, only two LTRs were elicited, which could have influenced these results.

## Study limitations

The current study has some limitations. First, treatment was only performed in the upper trapezius muscle. Although this muscle is a common source of neck pain, additional muscles could also have been evaluated. Second, as there was no placebo group, we cannot exclude a possible placebo effect of PENS or DN. Third, in this study PENS was applied for 15 min; however, doses greater than 20 min have been reported to produce analgesic effects. It would be interesting to apply greater doses of PENS in future studies.

## Conclusion

The application of PENS after DN showed greater short-term improvements in post-needling soreness, neck pain, and mechanical hyperalgesia than DN alone, which leads to the conclusion that the combination of PENS and DN is more beneficial immediately following treatment than applying DN alone in patients with chronic myofascial neck pain. Therefore, PENS could be recommended in order to reduce post-needling soreness after the application of deep dry needling.
